# Prophylactic options in patients with 5-fluorouracil-associated cardiotoxicity

**DOI:** 10.1038/sj.bjc.6600967

**Published:** 2003-05-13

**Authors:** G Cianci, M F Morelli, K Cannita, R Morese, E Ricevuto, Z C Di Rocco, G Porzio, P Lanfiuti Baldi, C Ficorella

**Affiliations:** 1 Department of Medical Oncology, S. Salvatore Hospital, University of L'Aquila, 67100 L'Aquila, Italy; 2 Department of Experimental Medicine, University of L'Aquila, Via Vetoio, Coppito II, L'Aquila, 67100 Italy

**Keywords:** prophylaxis, cardiotoxicity, 5-fluorouracil

## Abstract

At present, the various mechanisms involved in 5-fluorouracil (5-FU)-correlated cardiotoxicity remain to be elucidated and a universally accepted prophylaxis or treatment for this specific toxicity is not available. Although it may improve time to progression, survival and clinical benefit, a 5-FU-based regimen usually has to be discontinued if a patient experiences cardiotoxicity. Here, we describe our experience with three cases of 5-FU-associated cardiotoxicity. The angina-like pain that appeared approximately 95 h after beginning 5-FU therapy was apparently independent of the drug's administration modality. In the two patients receiving 5-FU 12-h flat continuous infusion from 22.00 to 10.00 h (5-FU 12-h c.i.) in combination with other drugs, the dose of 5-FU was reduced by 10–20% and patients received prophylactic transepidermal nitroglycerin. In the third patient, 5-FU administration modality was changed and prophylactic therapy was not given. By taking these precautions, the patients no longer complained of anginal pain and none of them discontinued chemotherapy.

5-Fluorouracil (5-FU) is an active antimetabolite used in several tumours, such as gastrointestinal tumours, in which the choice of effective alternative drugs is limited (Kohne *et al*, 1998). The spectrum of toxicities associated with 5-FU involves the gastrointestinal tract, the bone marrow, the skin and the central nervous system. Last, but not the least, 5-FU may give cardiac toxicity that is less familiar to physicians and this may present as precordial pain, atrial arrhythmias, electrocardiogram (EKG) ST-T wave changes, ventricular dysfunction and cardiogenic shock.

The overall incidence of 5-FU cardiotoxicity varies from 1.2 to 18% of patients and is usually underestimated, since silent EKG alterations often occur (Klaus Becker *et al*, 1999). The different mechanisms involved in 5-FU-associated cardiotoxicity are not yet fully understood and no unequivocally effective prophylaxis or treatment for this specific toxicity exists. Here, we describe three cases of 5-FU-associated cardiotoxicity, its clinical management and the prophylactic treatment used.

## PATIENTS AND METHODS

### Case 1

A 57-year-old male, with positive family history for acute myocardial infarction (AMI) but with no history of cardiovascular disease, came to our observation after undergoing an anterior resection of the rectum for a stage III rectal–sigmoid adenocarcinoma (RSA) (Table 1

**Table 1:** Clinical and pathological features of patients

	Case 1	Case 2	Case 3
Familiarity	AMI	—	—
Age	57	65	70
Clinical history	—	HBP, diabetes, gastritis	HBP
Tumour size	RSA	Ampulla of Vater	RSA
Phase	Metastatic	Metastatic	Adjuvant
Adjuvant therapy	No	No	Yes
Type	—	—	5-FU
Procedure	—	—	Bolus
Advanced therapy	Yes	Yes	No
Type	CPT11/5-FU	CDDP/5-FU	—
Procedure	Bolus/continuous infusion	Bolus/continuous infusion	—
Symptom	Chest pain	Chest pain	Chest pain
Duration	Few minutes	5 min	Few minutes
Cycle	1°	1°	3°
Day	4°	4°	4°
Therapy	Yes	Yes	Yes
Type	Nitroglycerin	Nitroglycerin	Nitroglycerin
Examinations	Yes	Yes	Yes
EKG	Negative	Ischaemic alteration	Negative
EchoCG	No	Reduction of contractility	No
Enzymes	Negative	Not done	Negative
Effort-EKG	No	Ventricular extrasystole	Negative
Other cycles	Yes	Yes	Yes
Number	5	6	3
Procedure	Bolus/continuous infusion	Bolus/continuous infusion	Bolus
Therapy	Nitroglycerin/dose reduction of 5-FU	Nitroglycerin/dose reduction of 5-FU	Weekly therapy
Other symptoms	No	No	No
Other chemotherapy	Yes	No	No
Type	OXP/5-FU	—	—
Procedure	Bolus/continuous infusion	—	—
Symptoms	No	—	—
Other cycles	Yes	—	—
Number	6	—	—
Procedure	Bolus/continuous infusion	—	—
Therapy	Nitroglycerin	—	—

AMI=acute myocardial infarction; CPT11=irinotecan; EKG=electrocardiogram; echoCG=echocardiogram; CDDP=cisplatin; OXP=oxaliplatin; 5-FU=5-fluorouracil; HBP=high blood pressure; RSA=rectal–sigmoid adenocarcinoma.

). Physical examination, chest X-rays, blood pressure and EKG were all within normal limits. The patient received four cycles of adjuvant chemotherapy with 5-FU (370 mg m^−2^ day^−1^) and folinic acid (FA) (50 mg tot^−1^) i.v. for 5 days, every 3 weeks.

During adjuvant chemotherapy, disease progressed with liver metastasis and the patient's therapy was changed to irinotecan (180 mg m^−2^) day 1 and 5-Fu 12-h flat continuous infusion from 22.00 to 10.00 h (5-FU 12-h c.i.) (900 mg m^−2^ day^−1^) for 4 days, every 2 weeks. Approximately 93 h after starting the first cycle of the second 5-FU regimen, the patient presented angina-like pain that lasted a few minutes and disappeared with oral nitroglycerin. The blood pressure was 160/115 mmHg and the heart rate was 105 beats min^−1^ (b.p.m.). During the anginal attack, EKG recordings and serum cardiac enzyme measurements were normal; therefore, the same chemotherapy infusion schedule was continued, but the dose of 5-FU was reduced to 800 mg m^−2^ day^−1^ and transepidermal nitroglycerin prophylaxis was administered during 5-FU 12-h c.i. The patient then received a further five cycles of this treatment without any chest pain. Upon disease progression, the patient received six cycles of oxaliplatin (100 mg m^−2^) day 1 and 5-FU 12-h c.i. (800 mg m^−2^ day^−1^) for 4 days, every 2 weeks always associated with prophylactic transepidermal nitroglycerin. No angina-like syndrome recurred and no EKG changes were noted.

### Case 2

A 65-year-old male with high blood pressure (HBP), diabetes and gastritis came to our attention with abdominal lymphatic and pulmonary metastases from an ampulla of Vater adenocarcinoma (Table 1). He received one cycle of induction chemotherapy containing cisplatin (CDDP) (100 mg m^−2^) day 1 and 5-FU 12-h c.i. (1000 mg m^−2^ day^−1^) for 5 days, every 3 weeks. Approximately 95 h after beginning the first cycle of 5-FU infusion, the patient referred several episodes of anginal pain, associated with sweating, that disappeared with oral nitroglycerin. On day 5 of cycle I, the blood pressure was 130/80 mmHg, the heart rate was 104 b.p.m. and the EKG recorded shortly after pain remission showed ischaemic alterations. The patient was admitted to the hospital and immediately subjected to an echocardiogram that revealed a reduction in inferior and posterolateral contractility and an altered left ventricle diastolic release with an ejection fraction of 56%. The EKG recorded the following day showed a T-alteration in the anterolateral leads and a nonspecific ST segment elevation in the inferior leads. At 4 days after the pain episode, the EKG showed a T-alteration in the lateral leads, likely due to an AMI with ischaemic alterations. The effort-EKG obtained on the same day was interrupted at 80% of the maximum because ventricular extrasystole bigeminis rised. The patient was subjected to CT-scan for restaging of disease. This showed a 50% reduction of pulmonary metastases and disappearance of abdominal lymphatic metastasis. For this reason, the same chemotherapy infusion schedule was continued, but the dose of 5-FU was reduced to 800 mg m^−2^ day^−1^. Transepidermal nitroglycerin was administered during 5-FU infusion and chemotherapy was administered while the patient recovered at the hospital. The dose of 5-FU for the cycles thereafter was increased to 900 mg m^−2^ day^−1^ with transepidermal nitroglycerin administration during 5-FU infusion. The patient received a further four cycles of this treatment, in an outpatient setting, and no longer complained of anginal pain.

### Case 3

A 70-year-old female with HBP presented at our hospital with stage II RSA (Table 1). She received three cycles of adjuvant chemotherapy with bolus 5-FU (370 mg m^−2^ day^−1^) and FA (50 mg tot^−1^ day^−1^) for 5 days every 3 weeks. On the third cycle, approximately 98 h after starting 5-FU administration, the patient presented a transient angina-like pain with dyspnoea that disappeared with oral nitroglycerin. The EKG did not reveal specific changes, plasma cardiac enzymes and the effort-EKG were normal. 5-Fluorouracil therapy was not discontinued but the schedule was modified to 5-FU 450 mg m^−2^ week^−1^ and FA 100 mg tot^−1^ week^−1^ for 14 weeks; although transepidermal nitro-glycerin prophylaxis was not given during 5-FU administration, the patient no longer complained of anginal pain.

## DISCUSSION

**Figure 1:**
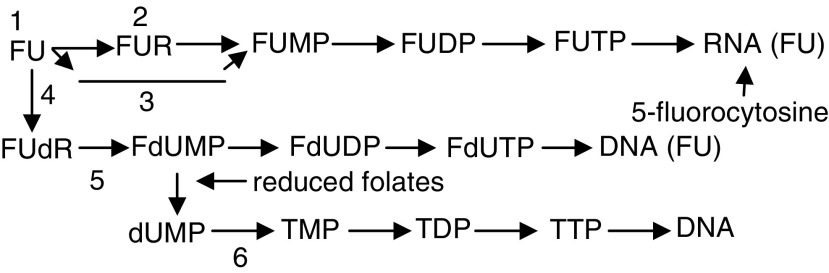
Fluorouracil metabolism: FUR=fluorouridine; FUMP=fluorouridine-monophosphate; FUDP=fluorouridine-diphosphate; FUTP= fluorouridine-triphosphate; dU=deoxyuridine; 1=uridine phosphorylase; 2=uridine kinase; 3=orotate phosphoribosyltransferase; 4=thymidine phosphorylase; 5=thymidine kinase; 6=thymidylate synthetase.

Relatively S-phase specific, 5-FU is a synthetic pyrimidine antimetabolite drug that has been used as a cytostatic agent in the treatment of various solid malignant tumours (adenocarcinomas, squamous cell cancer). The antitumour activity of 5-FU is exerted through several mechanisms of action and its dose, route of administration and administration schedule may play a critical role in its mechanism of action. Initially, 5-FU is inactive and is converted, within cells, into various active nucleotide forms (Rose *et al*, 2002): 5-fluoro-2′-deoxyuridine-5′-monophosphate (FdUMP) is one of the critical nucleotide metabolites generated in tumour cells and other sensitive tissues (Figure 1

).

FdUMP potently inhibits thymidylate synthetase by competitive binding, resulting in a thymine-depleted state (‘thymine-less death’). This is enhanced by folate cofactors. Cytotoxic effects are also mediated through the incorporation of fluorodeoxyuridine triphosphate into DNA (seen in continuous 5-FU infusion), and of fluorouridine-5′-triphosphate (FUTP) and 5-fluorocytosine into RNA (seen in bolus 5-FU). Furthermore, these metabolites are also thought to alter membrane function (calcium channels), interfere with mitochondrial metabolism (energy failure phosphate balance), inhibit ribosomal RNA, alter macromolecule characteristics (contractile elements), cause oxidative damage (pyrimidines are thought to play a role in favism) and release of vasoactive substances (histamine and catecholamines are known to cause ultrastructural changes similar to doxorubicin toxicity) and induce autoimmune activity (complexes between 5-FU cells or exposure of immunogenic native compounds after cell damage) (Norwood *et al*, 1993).

The postulated causative mechanisms involved in 5-FU cardio-toxicity are the following: an autoimmune response to damaged cells; an increased oxygen demand in patients receiving 5-FU; a coronary spasm caused by protein kinase C-mediated vasoconstriction; dihydropyrimidine dehydrogenase deficiency (Milano *et al*, 1999) and the 5-FU contaminant fluoroacetate. Inhibition of DNA synthesis, due to 5-FU incorporation into myocardial cells, was suggested to be the first step of cardiotoxicity and myocardial depression has been explained by inhibition of mitochondrial DNA synthesis due to 5-FU (Kohne *et al*, 1998). Furthermore, it has been demonstrated that 5-FU may cause damage to endothelial cells with consequent thrombus formation. In our experience, 5-FU-associated effects on vascular endothelium reached their peak about 3 days from initiation of treatment, which corresponds to the clinical course of 5-FU cardiotoxicity (Cwikiel *et al*, 1995,^1996^).

According to the analysis of 114 case reports, cardiotoxicity was experienced in 61 *vs* 33% of patients who received 5-FU 12-h c.i. or bolus 5-FU, respectively; 20% of the patients for whom data were available had a history of coronary artery disease or some other heart disease (Norbertus *et al*, 1993). Data do not support the conclusion that dose and mode of administration of 5-FU therapy may be an important factor in the development of cardiac toxicity (Norwood *et al*, 1993). Data do not support the conclusion that a history of cardiac disease increases the risks of 5-FU-induced cardiotoxicity (Norbertus *et al*, 1993). CDDP frequently is combined with 5-FU, and although a synergistic effect of CDDP or carboplatin with 5-FU cannot be excluded, 5-FU therapy alone could completely explain the toxicity observed. After the chest pain appears, 5-FU treatment is usually discontinued and the response to supportive treatment with nitrates or calcium-channel blockers is good (Norbertus *et al*, 1993).

Many authors suggest that prophylaxis with cardioprotective agents, after development of 5-FU-associated coronary ischaemia, permits protracted 5-FU administration, where clinically indicated (Oleksowicz and Bruckner, 1988; Norwood *et al*, 1993).

According to our experience, the average age of patients was 64 years, none had a history of cardiac disease, but two patients had HBP. Two patients were treated with combination therapy regimens. Apparently, 5-FU administration modality did not influence the angina-like pain presentation that appeared approximately 95 h after starting 5-FU therapy. The angina-like syndrome disappeared with oral nitroglycerin and by stopping infusion.

Since it was deemed necessary, 5-FU treatment was not discontinued in our patients. For the two patients (Cases 1 and 2) receiving nocturnal 5-FU infusion in combination therapy, the dose of 5-FU was reduced by 10–20% and patients were given transepidermal nitroglycerine prophylaxis. 5-Fluorouracil administration modality was changed in the third patient (Case 3): the bolus schedule (5-FU 370 mg m^−2^ day^−1^ plus FA 50 mg tot^−1^ day^−1^ for 5 days every 3 weeks) was modified to a weekly schedule (5-FU 450 mg m^−2^ week^−1^ and FA 100 mg tot^−1^ week^−1^), without prophylactic therapy.

By taking the above-mentioned precautions, patients continued therapy and no longer complained of acute angina-like pain.
